# Supplementary material for: Solanum lycopersicum Seedlings. Metabolic Responses Induced by the Alkamide Affinin
Source: Metabolites. 2021 Feb 27;11(3):143. doi: 10.3390/metabo11030143 (PMC7997251; doi:10.3390/metabo11030143)
Supplement: Supplementary file 1 [file metabolites-11-00143-s001.pdf]

Table S1. Retention times (RT), Metabolite, trimethylsilyl derivative (TMS) and Peak Purity used for identification of the compounds detected in shoots and roots of tomato seedlings by GC/EIMS.

ID	RT (min)	Metabolite	TMS	Peak Purity	Shoot	Root	ChEBI ID	Category
1	13.3040	Ethylamine	2TMS	90	+	+	15862	Others
2	16.4028	Lactate	2TMS	96	+	+	24996	Organic acid
3	16.7564	Glycolate	2TMS	74	+	+	29805	Organic acid
4	17.8287	Alanine	2TMS	92	+	+	15570	Amino acid
5	17.9175	Levulinic acid	1TMS	85	+	+	45630	Others
6	18.1660	Oxalate	2TMS	79	-	+	16995	Organic acid
7	19.7151	Phosphoric acid monomethyl ester	2TMS	98	+	+	340824	Others
8	19.8428	Cadaverine	4TMS	70	-	+	18127	Others
9	21.0792	3-Hydroxyisovalerate	2TMS	70	+	+	82957	Others
10	21.4034	Valine	2TMS	96	+	+	27266	Amino acid
11	21.6860	Urea	2TMS	93	+	+	16199	Others
12	22.8820	Phosphoric acid	3TMS	96	+	+	26078	Others
13	23.0354	Leucine	2TMS	84	+	+	25017	Amino acid
14	23.1025	Ethanolamine	3TMS	94	+	+	16000	Others
15	23.3084	Glycerol	3TMS	97	+	+	17754	Others
16	23.3435	Maleate	2TMS	87	+	+	18300	Organic acid
17	23.6710	Isoleucine	2TMS	80	+	+	27730	Amino acid
18	23.6837	Succinate	2TMS	90	+	+	15741	Organic acid
19	24.0794	Glycine	3TMS	96	+	+	15428	Amino acid
20	24.6081	Glycerate	3TMS	86	+	+	16659	Sugar acid
21	24.7252	Fumarate	2TMS	94	+	+	18012	Organic acid
22	25.0506	Pelargonic acid	1TMS	70	-	+	29019	Fatty acid
23	25.5311	Serine	3TMS	97	+	+	17822	Amino acid
24	26.3006	Threonine	3TMS	97	+	+	16398	Amino acid

25	27.2343	Beta-Alanine	3TMS	73	+	+	16958	Amino acid
26	27.5909	Ornithine-1,5-lactam	2TMS	82	+	-	76341	Others
27	27.8048	Homoserine	3TMS	70	+	-	30653	Amino acid
28	28.7035	Malate	3TMS	96	+	+	6650	Organic acid
29	29.2077	Pyroglutamic acid	2TMS	96	+	+	16010	Others
30	29.3062	Methionine	2TMS	60	+	+	16811	Amino acid
31	29.4650	Aspartate	3TMS	94	+	+	22660	Amino acid
32	29.8269	GABA	3TMS	84	+	+	16865	Amino acid
33	30.7013	Threonate	4TMS	72	-	+	15908	Sugar acid
34	31.7885	Glutamate	3TMS	86	+	+	16015	Amino acid
35	31.8988	Phenylalanine	2TMS	83	+	+	17295	Amino acid
36	32.2782	Lauric acid	1TMS	74	-	+	30805	Fatty acid
37	32.9159	Asparagine	3TMS	97	+	+	17196	Amino acid
38	35.1627	Glutamine	3TMS	93	+	+	18050	Amino acid
39	35.3133	Octopamine	3TMS	70	-	+	17134	Others
40	35.8866	Glycerate 3P	4TMS	70	-	+	17050	Phosphorylated compound
41	35.9753	Shikimate	4TMS	80	+	+	36208	Organic acid
42	36.3359	Isocitrate	4TMS	76	+	+	30887	Organic acid
43	36.5081	Ornithine	4TMS	82	+	-	15729	Amino acid
44	37.0650	Fructose	5TMS	94	+	+	28757	Sugar
45	37.9558	Histidine	3TMS	70	+	-	15971	Amino acid
46	38.5760	Tyrosine	3TMS	70	+	-	17895	Amino acid
47	38.5878	Lysine	4TMS	75	-	+	18019	Amino acid
48	39.0548	Glucose	5TMS	97	+	+	17634	Sugar
49	40.3479	Palmitic acid	1TMS	92	+	+	15756	Fatty acid
50	40.5495	Gluconate	6TMS	70	+	+	86359	Sugar acid
51	40.8616	Galactarate	6TMS	86	+	+	30852	Sugar acid
52	42.3888	Myo-Inositol	6TMS	97	+	+	17268	Others

53	42.8462	Phytol	1TMS	78	+	-	17327	Others
54	43.2458	Linoleic acid	1TMS	90	+	+	17351	Fatty acid
55	43.3708	Oleic acid	1TMS	75	-	+	16196	Fatty acid
56	43.8634	Stearic acid	1TMS	54	+	+	28842	Fatty acid
57	44.8401	Fructose-6-Phosphate	6TMS	80	+	+	78697	Phosphorylated compound
58	45.1549	Mannose-6-phosphate	6TMS	72	+	+	17369	Phosphorylated compound
59	46.0508	Chlorogenic acid	6TMS	85	+	+	16112	Others
60	46.8596	Glucose-6-phosphate	6TMS	91	-	+	14314	Phosphorylated compound
61	47.0775	Arachidic acid	1TMS	34	+	+	28822	Fatty acid
62	47.8294	Myo-inositol-1-phosphate	7TMS	70	-	+	18297	Phosphorylated compound
63	49.7481	1-Monopalmitoylglycerol	2TMS	86	+	+	69081	Fatty acid
64	52.5342	Sucrose	8TMS	93	+	+	17992	Sugar
65	53.8960	Glycerol monostearate	2TMS	93	+	+	75555	Fatty acid
66	59.2775	Beta-Melibiose	8TMS	96	+	+	28053	Sugar
+ Detected; - No detected								

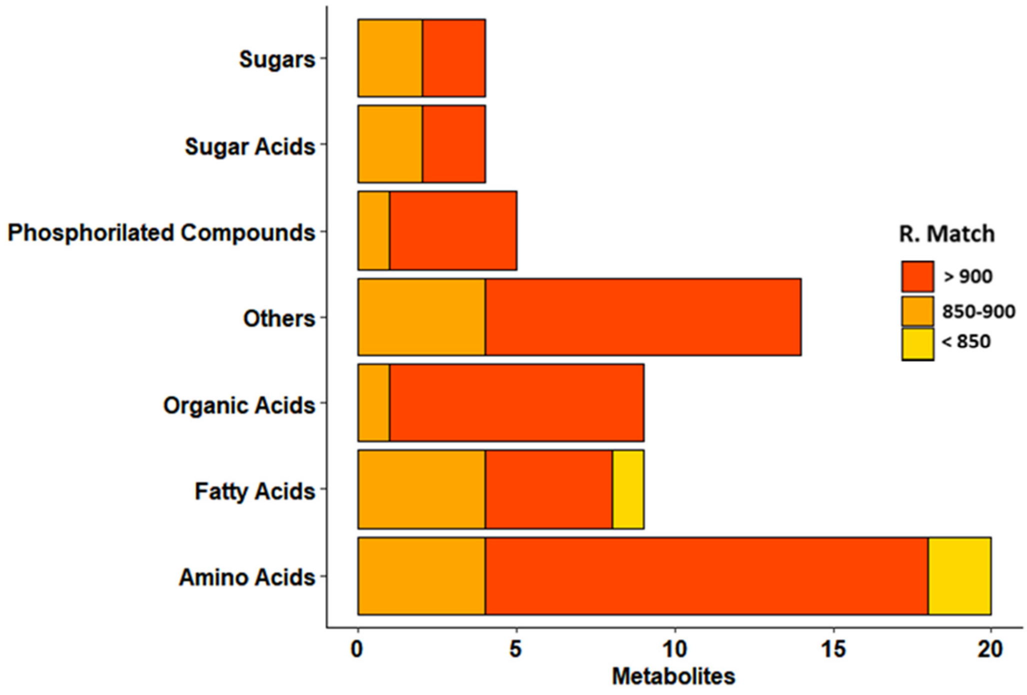

Figure S1. Categorization of detected metabolites. Y axes represent the number of metabolites while X is the category. The bars are stacked by the R. Match value of each detected metabolite. It can be seen that most R. Match values are> 850 and only a very few are below 850.

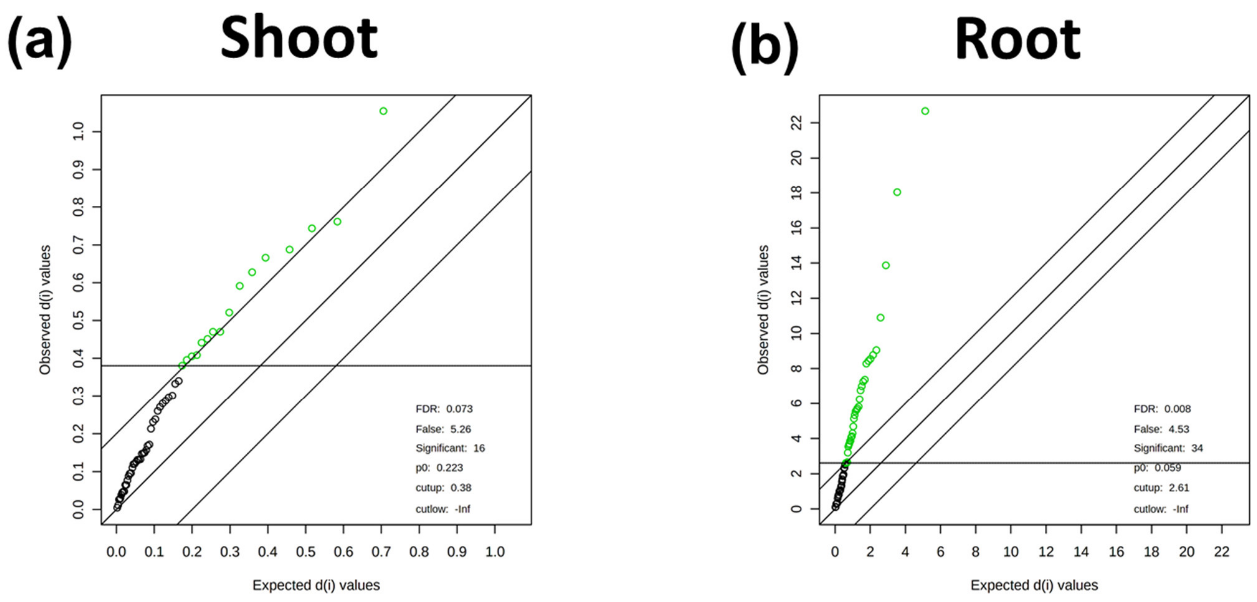

Figure S2. Significance Analysis of Microarrays SAM to detect metabolites with significant relative abundances in response to affinin. . **(a)** Shoots thirteen and **(b)** Roots thirty-seven metabolites with statistical differences. Delta is the distance between the observed and the expected (ordered) test scores. Plot shows the observed relative difference versus the expected relative difference estimated by data permutation. The horizontal lines are the delta value (0.2 for shoots and 2.0 for roots). The solid diagonal line indicates where these two measures are the same. The significant features are highlighted in green.

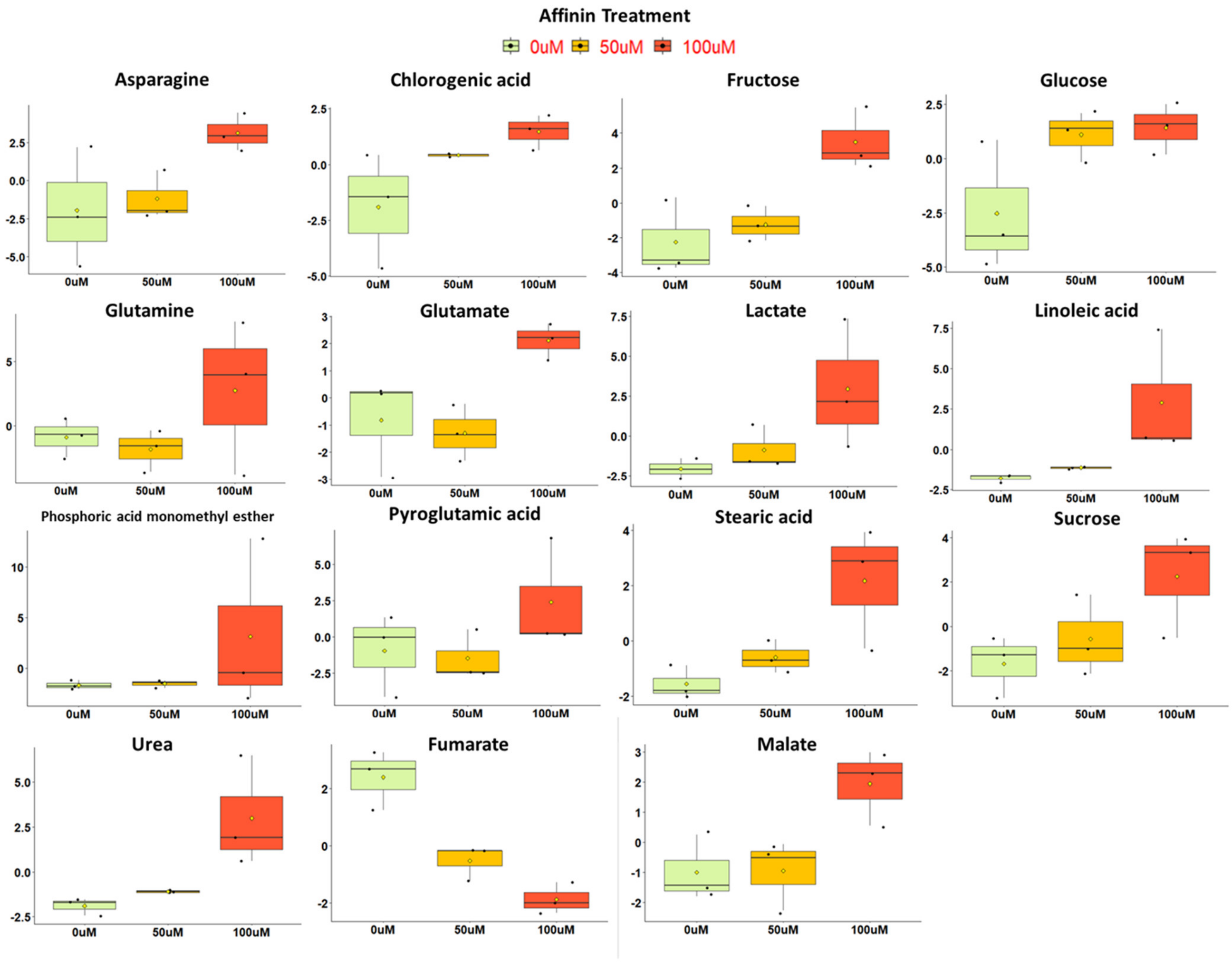

Figure S 3 Box plot of the normalized relative abundances of the thirteen metabolites detected with the SAM analysis. Data were normalized by dividing the compound peak area by fresh weight of sample, then transformed with the cube root transformation and scaled by Pareto algorithm. Due to this process, we obtained negative scale in the Y-axis in some of the bins (Metaboanalyst 4.0). The bar plots show the normalized values (mean +/- one standard deviation). The boxes range from the 25% and the 75% percentiles; the 5% and 95% percentiles are indicated as error bars. Single data points are indicated by black circles. Medians are indicated by horizontal lines within each box and means are indicated by a yellow diamond.

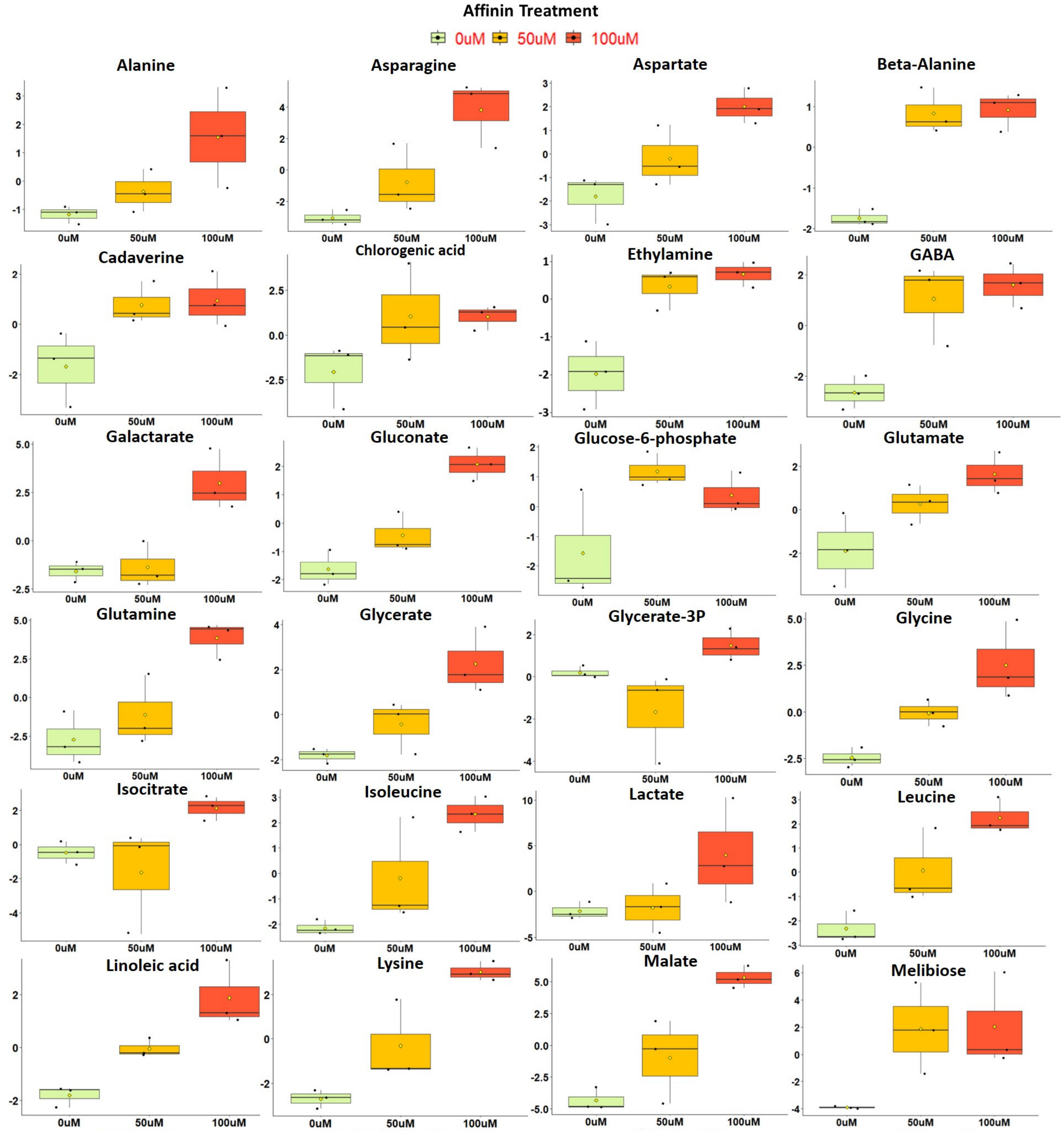
Affinin Treatment
0uM
50uM
100uM
Alanine
Asparagine
Aspartate
Beta-Alanine
Cadaverine
Chlorogenic acid
Ethylamine
GABA
Galactarate
Gluconate
Glucose-6-phosphate
Glutamate
Glutamine
Glycerate
Glycerate-3P
Glycine
Isocitrate
Isoleucine
Lactate
Leucine
Linoleic acid
Lysine
Malate
Melibiose

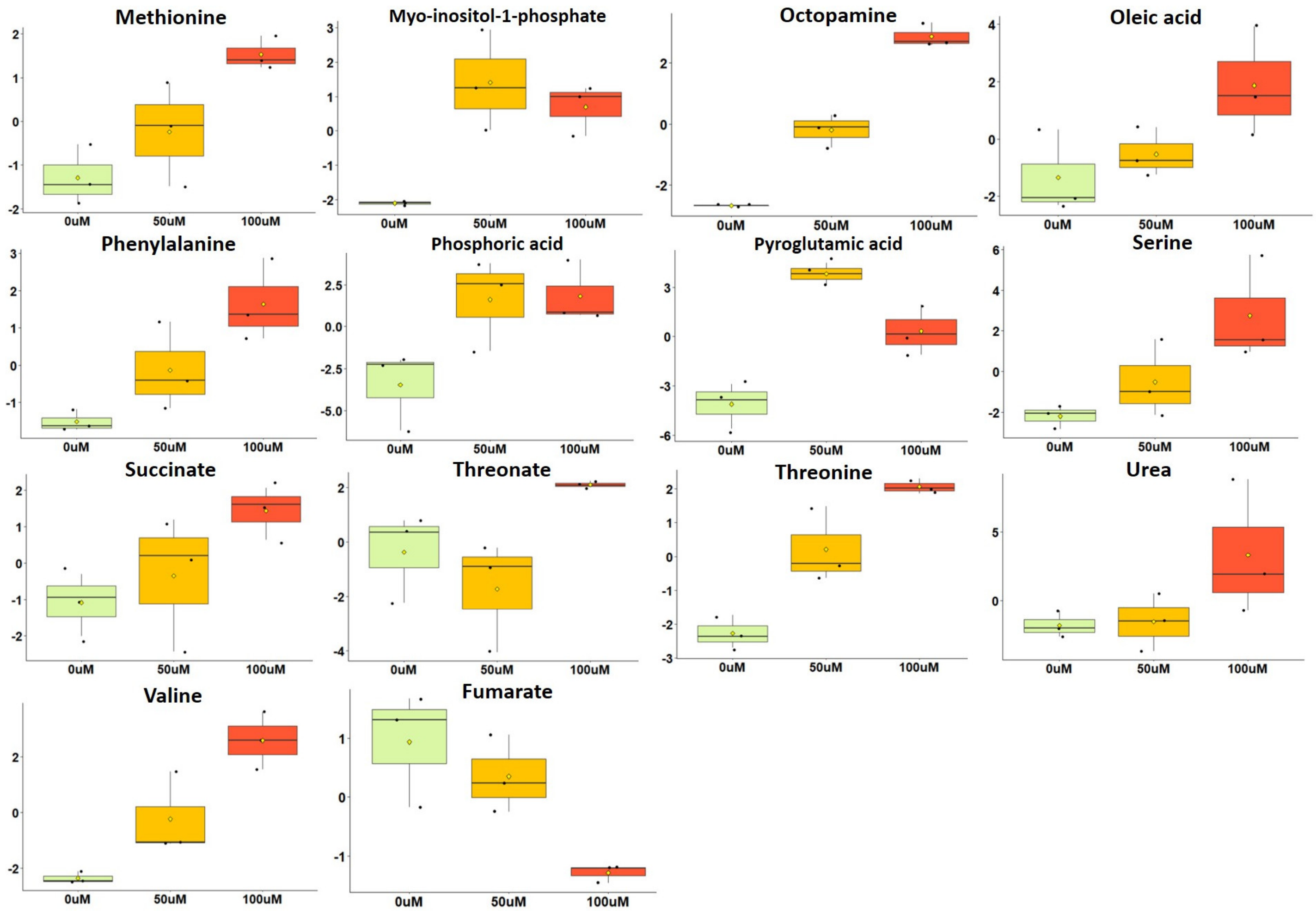

Figure S 4. Box plot of the normalized relative abundances of the thirty-seven metabolites detected with the SAM analysis. Y axes are represented as normalized relative abundances. Data were normalized by dividing the compound peak area by fresh weight of sample, then transformed with the cube root transformation and scaled by Pareto algorithm. Due to this process, we obtained negative scale in the Y-axis in some of the bins (Metaboanalyst 4.0). The bar plots show the normalized values (mean +/- one standard deviation). The boxes range from the 25% and the 75% percentiles; the 5% and 95% percentiles are indicated as error bars. Single data points are indicated by black dots. Medians are indicated by horizontal lines within each box and means are indicated by a yellow diamond.
